# COVID-19 Intelligence-Driven Operational Response Platform: Experience of a Large Tertiary Multihospital System in the Middle East

**DOI:** 10.3390/diagnostics11122283

**Published:** 2021-12-06

**Authors:** Osama A. Alswailem, Bashar K. Horanieh, Arwa AlAbbad, Sarab AlMuhaideb, Abdulkarim AlMuhanna, Muhammad AlQuaid, Hisham ElMoaqet, Nuhad Abuzied, Ahmad AbuSalah

**Affiliations:** 1 Healthcare Information Technology Affairs, King Faisal Specialist Hospital & Research Center, Riyadh 11211, Saudi Arabia; alswailem@kfshrc.edu.sa (O.A.A.); bashar@kfshrc.edu.sa (B.K.H.); malquaid@kfshrc.edu.sa (M.A.); nuhad@kfshrc.edu.sa (N.A.); 2 Center for Healthcare Intelligence, Healthcare Information Technology Affairs, King Faisal Specialist Hospital & Research Center, Riyadh 11211, Saudi Arabia; aalabbad@kfshrc.edu.sa (A.A.); muhanna@kfshrc.edu.sa (A.A.); 3 Computer Science Department, College of Computer and Information Sciences, King Saud University, Riyadh 11451, Saudi Arabia; almuhaideb@ksu.edu.sa; 4 Department of Mechatronics Engineering, School of Applied Technical Sciences, German Jordanian University, Amman 11180, Jordan

**Keywords:** hospital operations, decision support, COVID-19, visualization, advanced analytics, artificial intelligence

## Abstract

The COVID-19 pandemic has resulted in global disruptions within healthcare systems, leading to quick dynamic fluctuations in hospital operations and supply chain management. During the early months of the pandemic, tertiary multihospital systems were highly viewed as the go-to hospitals for handling these rapid healthcare challenges caused by the rapidly increasing number of COVID-19 cases. Yet, this pandemic has created an urgent need for coordinated mechanisms to alleviate increasing pressures on these large multihospital systems and ensure services remain high-quality, accessible, and sustainable. Digital health solutions have been identified as promising approaches to address these challenges. This case report describes results for developing multidisciplinary visualizations to support digital health operations in one of the largest tertiary multihospital systems in the Middle East. The report concludes with some lessons and insights learned from the rapid development and delivery of this user-centric COVID-19 multihospital operations intelligent platform.

## 1. Introduction

The continuously varying global COVID-19 pandemic situation challenged the maturity and resilience of hospitals’ existing digital and analytics infrastructure [1,2,3]. Particularly, it challenged the role of data as a strategic asset for informing decision-making processes [1,2,3]. This was clearly pronounced in the healthcare sector through the lack of accurate, timely, and readily available data-driven insights that reflect the actual situation awareness for operating units in hospitals [4,5,6].

Tertiary multihospital systems are highly viewed as the go-to hospitals for handling these challenges since it is equipped with the needed specialists, facilities, and supplies to absorb the rapidly increasing number of COVID-19 cases [1,2,3]. They are also equipped with capabilities to re-organize their usual activities and classical structure to meet the increasing demand for hospitalization in COVID-19 patients [7,8]. Accordingly, the tertiary multihospital systems quickly realized the urgency to have handy tools for informing day-to-day operations management and planning activities [1,4,5,6]. The tools shall automatically synthesize and produce a truly holistic view of current and future system capacity, utilization, and needs from temporal and spatial perspectives [4,5,9]. King Faisal Specialist Hospital and Research Centre (KFSH&RC) is among the largest tertiary multihospital systems in the Middle East region. Its main facilities are in Riyadh, Jeddah, and Madinah within the Kingdom of Saudi Arabia (KSA). Combined, the hospital has more than 1800 beds and 14,000 staff and clinical consultants. On average, it has 30,000 admissions and 90,000 ER visits on an annual basis. From a digital and analytics infrastructure perspective, the hospital achieved HIMSS Analytics Electronic Medical Record Adoption Model (EMRAM) Level-7 [10], HIMSS Analytics Adoption Model for Analytics Maturity (AMAM) Level-6 [11], HIMSS DAVIES Healthcare Service Excellence Award [12], the College of Healthcare Information Management Executives (CHIME) digital Health Most Wired Hospital Award [13], and many other achievements that reflect a very high level of digital and data infrastructure maturity for supporting KFSH&RC strategic goal of achieving excellence in care.

On 9 March 2020 KFSH&RC established the COVID-19 Command and Control Center to serve as an enterprise-wide governance and executive body for managing all COVID-19 related issues. It consists of hospital executives, operational leaders, and domain experts. Its main responsibility is to deploy effective COVID-19 strategic and tactical preparedness and response plans that cover patient care protocols, capacity preparation, testing and isolation facilities, as well as staff and supplies availability. Within the Kingdom of Saudi Arabia, the first COVID-19 patient was identified on 2 March 2020 [14]. Over the year 2020, the capital Riyadh as well as the coastal city of Jeddah, were leading other cities in number of COVID-19 cases [15].

The KFSH&RC COVID-19 Command and Control Center needed an enterprise-wide holistic situation awareness view of the hospital operations through a handy and easy to use set of tools. The situation awareness system shall reflect the actual current status as well as projected future care demands by leveraging disparate national and hospital data sources. It shall mainly cover hospital operations demands related to supplies and utilization. To address this urgent need, a multidisciplinary development team was formed with experts from across the enterprise including leadership, operational units, informatics, research, application development and advanced analytics. The team had the capabilities required for this effort based on their education, skills, and experience. The team also leveraged the valuable lessons learned from the hospital previous experience in managing the Middle East Respiratory Syndrome CoronaVirus (MERS-CoV) outbreak in 2015–2017 in Saudi Arabia [16,17,18].

This case report describes the methods and results for the development of a data-driven user-centric COVID-19 multihospital operations intelligent platform in KFSH&RC. The development process leveraged a 3-phase Situation Awareness (SA) model to identify the system stakeholders along with their corresponding needs, which were focused on comprehending current and projected COVID-19 care demands. The report concludes with some lessons and insights learned from the development and delivery of this COVID-19 operations intelligence platform. The delivered platform went live in April 2020.

## 2. Materials and Methods

### 2.1. Stakeholders & Need Analysis

An early alignment among various groups on needs, goals, and expectations is essential before starting the development process. This is very challenging, yet crucial, for comprehending COVID-19 care demands in a multihospital system due to several reasons: (1) Lack of established guidelines or recommendations for handling COVID-19 patients, (2) Need to interview a large group of individuals at various levels of power and interest with potential conflicting requirements within the system, and (3) Hard to avoid redundant interviews with stakeholders of same exact role but on different hospital locations [19,20,21]. For this work, a quick stakeholder analysis exercise was conducted by interviewing the members of the enterprise-wide COVID-19 Command and Control Center to clarify the project scope and impacted end users. Subsequent evaluations of proper stakeholder inclusion were also needed to focus on a granular sub-component of the project (e.g., operational unit). Then, we utilized the 3-phase Situation Awareness (SA) model to capture stakeholders’ needs and expectations, identify data sources, and design and develop visualizations [22,23]. The first phase (perception) of the SA model captures current COVID-19 care demands through collection of basic patients- and service-level temporal and spatial data. The second phase (comprehension) extracts information and knowledge from collected data using multi-dimensional data integration and analysis pipelines. The third phase (projections) projects future COVID-19 care demands through both advanced analytics and machine learning algorithms. The present case report covers these three phases of the SA model.

### 2.2. Development Methodology

The Rapid Application Development (RAD) approach was leveraged for software development given the urgent need of the stakeholders [24]. RAD emphasizes the software and actual end user feedback for driving design changes rather than strict planning and requirements recording. It prioritizes rapid prototype releases and iterations of the dashboard to reduce development time and speed up delivery. This approach assumes high-level of collaboration among all stakeholders, which was a key implementation driver in our case. The development group consists of members from: (1) the business & healthcare informatics team, which has the domain knowledge on various used business systems (EMR, ERP, Pharmacy Information Systems, LIS, etc.). (2) Data Engineering & Analysis team, which manages the data feeds for pulling data from business systems. (3) Artificial Intelligence team, which develops high accuracy projections for COVID-19 care demands. (4) Dashboard Development team, which has experts in embedding visualizations, data summarization, and deployment of models in a user-friendly interface. Finally, (5) Data & Cyber Security team that ensures compliance with enterprise and national data governance and cyber security regulations to guarantee full protection of patients’ privacy and enterprise data assets.

### 2.3. Multi-Dimensional Data Sources

The dashboard leveraged disparate national-level, hospital-level, and patient-level data sources, as illustrated in Table 1. Integration of these unconnected and independent data sources is crucial, to reflect a timely holistic hospital COVID-19 operations view. At the national level, COVID-19 statistics were pulled from the Saudi Ministry of Health [15]. At the hospital-level, the enterprise-wide COVID-19 surge plan was leveraged to automatically identify the active surge stage and corresponding care demands needs including capacity, staff, and supplies. Clinical patient-level data, such as demographics, medications, labs, and visit details were collected from various information systems, such as Cerner Electronic Medical Records (EMR) and ScriptPro Medicine Automated Dispensing System [25]. The Oracle ERP system along with in-house developed systems were utilized to provide staff employment records, isolation status, and contact tracing information for a live update of sick hospital employees and others exposed to infected staff. Supply chain management data were also collected to reflect availability of various clinical equipment, Personal Protective Equipment (PPEs), and test kits.

## 3. Results

The stakeholder analysis exercise identified the initial list of stakeholders, which focused on those who are both highly influential and of interest across various operational units and leadership levels. This mainly included the members of the COVID-19 Command and Control Center Committee. The list of stakeholders was then expanded over time to include domain-specific managers and experts for identification of domain-specific needs as well as the interpretation of collected data elements. The collected stakeholders’ needs were then assigned to the relevant SA level as shown in Table 2.

To address the stakeholders’ needs, the development team designed automated data extraction and aggregation pipelines to query the data sources shown in Table 1. The data are pulled every 15 min to reflect the care demand status in multiple healthcare facilities over diverse geographic locations including the main facilities in Riyadh, Jeddah, and Madinah. Patients were classified based on their admission location and infection severity. The collected infection severity levels are: Asymptomatic, Mild, Moderate, Severe, and Uncategorized, which are defined according to the COVID-19 guidelines published by the Saudi Ministry of Health [26]. Admissions were categorized based on patient location and the use of ventilator into home, isolation facility, ICU, and non-ICU ward. Supplies along with consumption rates were captured as well. This includes PPE supplies (fit solutions, gloves, gown, isolation supply, N95 masks, skin preparation, surgical masks, virus swab, and wipes). It also includes COVID-19 test kits supplies such as: Extraction Test (Abbott), Assay Test (Roche, Alton, and Solgent), EZ1 Rapid Extraction Test, Rapid KIA Stat Test, Cephide Test, Roche Cobas Test, Abbott SARS-CoV-2 Test, BioFire Test, Co-Diagnostic Test. Most gadgets on the platform were backed up by drill down capability to reach the desired granularity without compromising patient confidentiality in compliance with hospital and national patient privacy regulations.

Like typical healthcare records, some data elements have high signal to noise ratio while others have some degree of uncertainty and data aberrations. Such data quality issues initially hindered the discovery and the inference of actionable data-driven insights. Data profiling and quality management tools along with manual domain expert reviews were leveraged to address these data quality issues. Data latency and throughput were carefully considered to ensure data integrity.

The stakeholders repeatedly stressed the need to build a scalable system. This includes the ability to integrate additional data sources and features given the rapid discoveries related to COVID-19 management. For that, the implementation leveraged system’s APIs, standardized codes (ICD10, etc.), and common database structures [27]. The stakeholders also focused on maximizing the utilization of existing enterprise digital infrastructure and data visualization capabilities to speed up the development process, enhance dashboard utility, and ensure quality user experience.

The development team leveraged the available data management and visualization capabilities in the organization to ensure smooth integration with the existing digital infrastructure. This includes IIS on a Windows Server platform, Oracle DB, Visual FoxPro. The system also uses common standards such as HTML, CSS, and Javascript, Bootstrap, JQuery, Chart.js, JSON, Python, and AJAX for visualization and web publishing.

**Figure 1:**
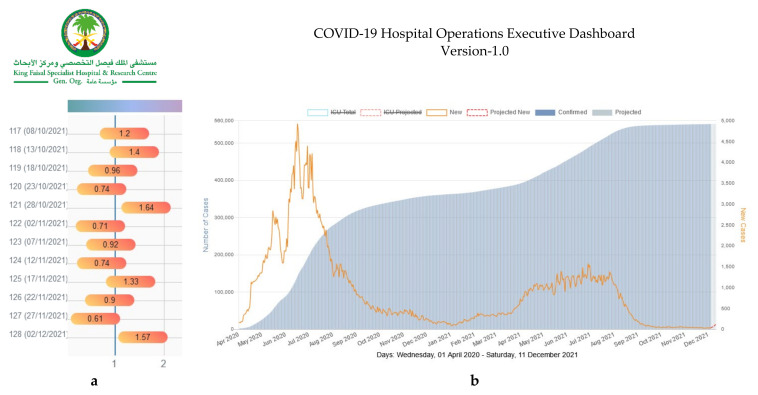
(**a**) National Effective R-value (5-day incubation period). (**b**) National Current and Projected COVID-19 Cases (5-day moving Average).

**Figure 2:**
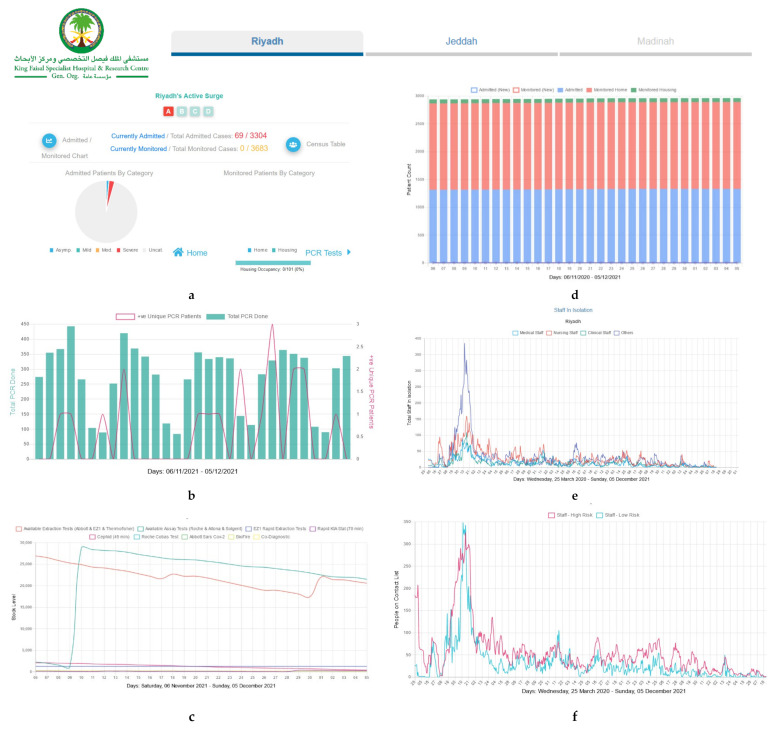
(**a**) Monitored patients per Severity level and Surge Level. (**b**) Number of PCR Tests Total and (+ve). (**c**) Monitored PCT test kits. (**d**) Patients Location. (**e**) Hospital staff in isolation. (**f**) Exposed staff as per contact list tracing.

**Figure 3:**
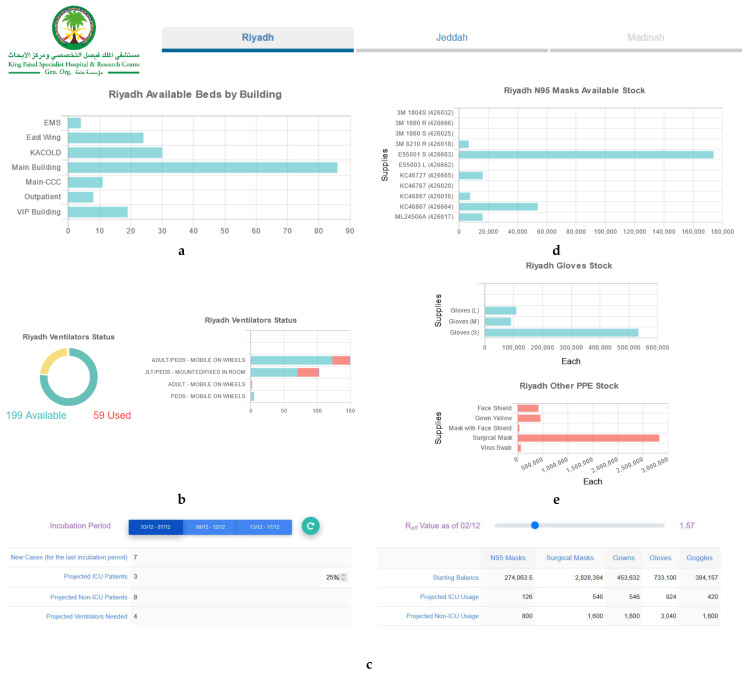
(**a**) Bed availability. (**b**) Ventilators’ utilization. (**c**) Projected PPE demands. (**d**) N95 Masks availability. (**e**) Other PPE supplies availability.

Figure 1 illustrates the visualization of the national-level insights of interest to the leadership group as per the identified needs demonstrated in Table 2. The visualizations in Figure 2 and Figure 3 address the needs of the leadership team as well as the operational leaders who are mainly focused on capacity management (beds, housing, sick and exposed staff, testing kits) across the various hospital locations. Figure 3 also shows the interactive multi-scenario projection tool that provides domain experts with a clear view of current and projected view of care demands and needs (PPEs, ICU beds, Non-ICU beds). The tool analyzes the trends of supplies and resources consumption at the hospital. It also leverages the guidelines and recommendations shared by the World Health Organization (WHO) and the US Centers for Disease Control and Protection (CDC) to inform future needs [28,29].

The dashboard has 54 active users of which 11 are executive leaders (members of the COVID-19 Command and Control Centre), 14 are operational leaders, and 29 are domain experts. It is used by the organization leadership during morning-huddles since March 2020. It has been also evaluated externally to ensure readiness, efficacy, and optimality of the business decisions made and addressed.

## 4. Discussion

The COVID-19 outbreak has created unprecedented challenges to the global healthcare system. Tertiary multihospital systems faced an elevated pressure due to the rapid growth of COVID-19 patient flow since it is equipped with the needed resources. Accurate management of resources is crucial to avoid issues pertaining to patient care and infection control. Therefore, a data-driven system that provides a holistic view of the hospital operations was rapidly developed to address the needs of hospital leaders and domain experts. The development process leveraged a 3-phase Situation Awareness (SA) model to identify the system stakeholders along with their corresponding needs. The needs are mainly focused on comprehending current and projected COVID-19 care demands. The needs were then used to drive the development of a COVID-19 operations intelligence platform for KFSH&RC.

After over one year of experience using the COVID-19 hospital operations intelligence platform, we identified some main key lessons learned that could be insightful for development teams undergoing a similar experience:

1. Leverage existing and available digital and analytics infrastructure capabilities of your organization to the full extent possible. This shall streamline the development process, enhance the user experience, and ensure smooth integration with various systems. At the beginning of the outbreak, there was a clear lack of readily available, accurate and easy to integrate off-the-shelf solutions. Various commercial solutions have become available over time, but the continuous need for customization and business updates are required and come at a steep unmanageable cost as the world’s knowledge and management of COVID-19 evolves.
2. Build standardized data feeds and pipelines that leverage code sets standards for conditions, lab tests, and other data sources. This is critical for system scalability, component reusability, and uniform data integration. This facilitates the rapid implementation of continuously changing stakeholders’ needs. It is also required for enabling effective data liquidity, which was a key design principal given the need for data to flow across various entities in the healthcare system.
3. The initial project stakeholders’ group shall include influential organization leaders with high interest in building operational intelligence platforms for the support of data-driven decision-making process. This group can quickly identify business priorities, top concerns, implementation timelines, other needed stakeholders, and scope of work. This will empower the implementation team to be fully aligned with actual organization needs. It also allows stakeholders to quickly illustrate the value and impact of the new system.
4. RAD methodology can speed up the delivery process of software development. A very strong collaboration between stakeholders and developers is required to ensure successful implementation of this methodology. The rapid prototyping and speedy end-user feedback were critical factors for enhancing productivity and illustrating system value at a shorter time period.
5. Building near real-time data feeds is better than real-time. While speed is important in operational intelligence, it is still acceptable to refresh your visualization every few minutes in lieu of seconds. This will reduce the load on your IT infrastructure and allows end users to comprehend the visualized data before being updated. For example, near real-time data showed good-enough insights for analyzing supplies consumption and staff availability.
6. Regularly maintain the system by dropping unneeded features, adding new data elements, and building nicer visualizations. This is critical as COVID-19 may have unpredictable future waves. Utilization of various systems features can be used as a key driver for future system enhancement.

## 5. Conclusions

This case report describes the methods as well as the results and main lessons learned from the rapid delivery of a user-centric COVID-19 multihospital operations intelligent platform in King Faisal Specialist Hospital and Research Center (KFSH&RC). A 3-phase Situation Awareness (SA) model was used to capture stakeholders’ needs and expectations, identify data sources, and design and develop multidisciplinary visualizations to support digital health operations in a large tertiary multihospital system in the Middle East. The developed system enabled smooth processing and integration for current COVID-19 care demands, as well as providing projections for future care demands through both advanced analytics and machine learning algorithms.

## Figures and Tables

**Table 1:** Dashboard Data Sources.

Data Type	System	Source/Description
National COVID-19 Statistics	National COVID-19 Data Repository	Maintained by the Saudi Ministry of Health. Provides live updates on national counts of COVID-19 cases and deaths.
Hospital COVID-19 preparedness planning data	Surge Plan	Developed by the COVID-19 Command Centre committee. Includes experts’ analysis of expected hospital needs (PPE, staff, rooms, etc.) per surge level.
Patient Electronic Medical Records	Cerner	Maintained by the Hospital IT & Analytics team. Includes all patient-level data such as: demographics, ordered labs and medications, PCR test results, health conditions, etc.
Supply Chain Management	Oracle ERP	Maintained by the Hospital IT & Analytics team. Includes all supply chain (consumption) data such as: test kits, equipment, medications, etc.
Human Resources (HR)	Oracle ERP	Maintained by the Hospital IT & Analytics team. Provides Human resources information about those patients who are also employees (staff and clinicians) at the hospital including their roles and units.
Dispensed Medications	ScriptPro	Maintained by the Hospital IT & Analytics team. Provides access to dispensed medications.
Isolated Staff and Contact Tracing	Home-grown System	Maintained by the Hospital IT & Analytics team. Provides access to staff in isolation and their contact tracing information list.

**Table 2:** Stakeholders Needs Analysis.

Needs Analysis		
Perception (SA Phase 1)	Comprehension (SA Phase 2)	Projection (SA Phase 3)
Goal:		
Simple representation of patient volume, service usage, supplies status.	Awareness of the current hospital capacity & operations.	Awareness of future care demands and hospital capacity.
Metrics (Per Location):		
COVID-19 Spread Rate. New, isolated, admitted, and ICU cases. Supplies (PPEs, test kits, etc.). Beds & rooms. Staff: nurses and physicians.	Patient volume growth. Supplies consumption. Staff, nurses, and clinicians’ availability. Service utilization.	Projected patient volume. Needed supplies. Services. Staff: nurses, and clinicians.
Design Consideration:		
Display collected data as counts or rates. Visualize collected data using simple charts and trends for comparative analytics.	Display hospital resources utilization using charts for comparative analytics. Automatically connect hospital resources utilization with the hospital surge plan.	Provide an interactive front end for end-users to generate multi-scenario projections based on various COVID-19 spread rates. Generate alerts which highlights possible future shortage of resources. Provide up to 3-weeks of projections.

## Data Availability

These may be obtained on request.
